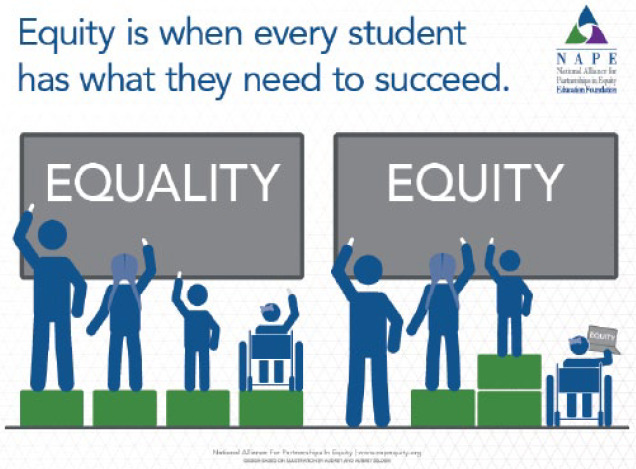# School Nurses: Living the Framework During COVID-19

**DOI:** 10.1177/1942602X20929533

**Published:** 2020-07

**Authors:** Laurie G. Combe

## A Novel Coronavirus Disrupts Our Way of Life

**Figure 1.**
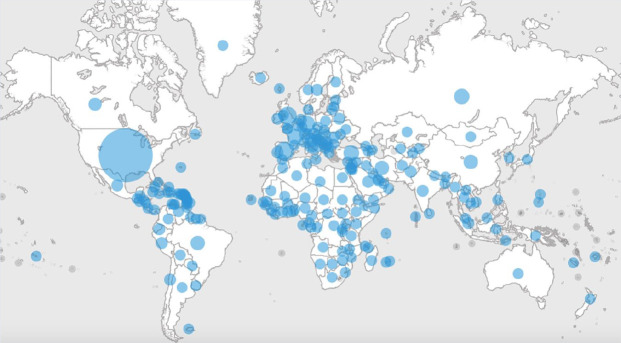
Coronavirus (COVID-19) (World Health Organization, May 5, 2020)

As I write this letter to you, it is late April and we are in the midst of an unprecedented event. While it has been just 4 months since we first learned of COVID-19 (Coronavirus Disease 2019), I feel like I have been taking this walk with you for a much longer time. Over the course of these months we have seen travel bans, physical distancing, rapid acceleration of case counts into the millions both worldwide and in the United States (see Figure 1), deaths in the hundreds of thousands (World Health Organization [WHO], 2020a), and brave healthcare providers on the frontline, often without the protection they need (American Nurses Association, 2020). Forty-three states have ordered school closures, affecting 55.1 million students (Education Week, 2020). The NASN2020 conference is “going virtual” in order to keep attendees and conference production staff safe and healthy.

**Figure 2.**
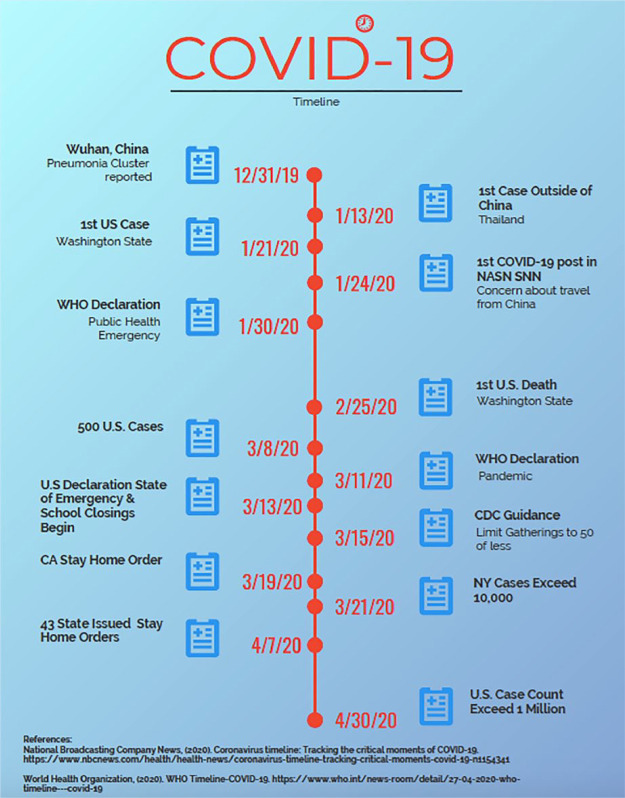
COVID-19 Timeline (NBC News, 2020; WHO, 2020b)

The pace of change in the COVID-19 response has been nothing short of frenetic (National Broadcasting Company News, 2020; WHO, 2020b). I regularly monitor the NASN All Member Forum School Nurse Net community and notice that NASN member Katie Morton (2020) sounded the first warning of the novel coronavirus on January 24 seeking guidance about travel from China into her community (see Figure 2). Since that time, all the School Nurse Net communities have logged almost 700 COVID-19-related posts and the conversation quickly turned to school reentry planning. By tracking the expressed needs of school nurses surrounding COVID-19, the NASN staff has worked tirelessly to build Coronavirus Disease 2019 Resources, including Return to School Guidance (NASN, 2020a).

### Managing and Adapting to Transitions

While schools were still open, early discussions in the school nurse community focused on the need for school nurses to access the same level of personal protective equipment (PPE) as that recommended for other healthcare providers (Centers for Disease Control and Prevention [CDC], 2020a, 2020b). School nurses discussed the evolving evidence surrounding COVID-19, considering how we could safely care for and isolate presumptive cases, manage aerosolizing procedures for ill or potentially asymptomatic students, and still provide safe care for the well population in our schools.

With schools closed, we are learning new ways to engage students in maintaining their health, while practicing within the constructs of ethical, legal, and professional nursing standards. School systems are relying on the expertise of school nurses to provide factual information about COVID-19; using their knowledge to develop plans that keep staff, students, and families healthy.

As nurses always do, we are looking ahead, anticipating the needs of students and school communities for that time when schools reopen again. School nurses are keeping pace with the evolving evidence, understanding that forecast model assumptions are based on yet to be determined factors such as the long-term viability of this novel coronavirus, policy decisions of political leaders, and citizen behavior (Michaud et al., 2020).

### Meeting Immediate Student Needs

School nurses know that many children face disparities in healthcare access, nutrition, safe housing, transportation, and more. Cindy Begley’s staff is making telephone calls to check on students with known health conditions, making sure that families have the resources they need to maintain health. Collaboration with school counselors provides Cindy’s staff with a resource list to meet needs that families are expressing.

**Figure 3.**
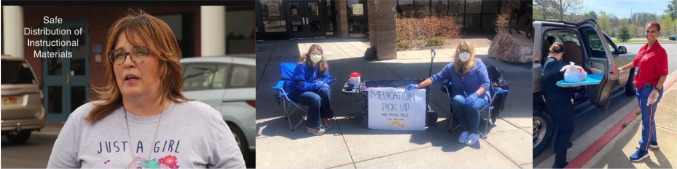
Amy Ponce; Angela Pesche and Kindra Schutt; Paulette Abbey

School nurse Amy Ponce is making sure that distribution of instructional materials is done in manner that protects the health of students, families, and staff (Figure 3). Because their schools will be closed for the remainder of this school year, Angela Pesche and Kindra Schutt are staffing a medication pickup to return supplies to families, making sure they have enough medication at home (Figure 3). Paulette Abbey works with her school food service partners to ensure that students dependent on school nutrition continue to have their needs met (Figure 3).

### Virtual School Nursing

**Figure 4.**
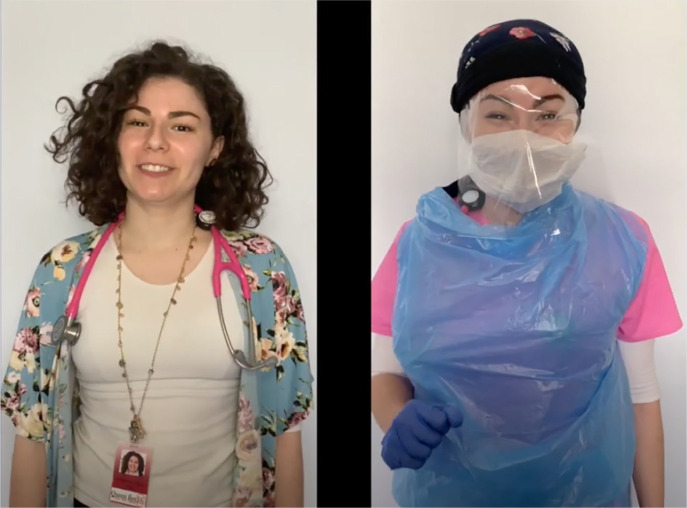
Jenna Palmisano

Schools nurses are experts at adapting our nursing practice to the school environment. As schools flipped to remote learning we hopped on board. School nurses are dropping by teacher’s remote classrooms to connect with students and collaborate with teachers to identify students at risk for chronic absenteeism. Jenna Palmisano (2020) produced a health education video, We Are Nurses!, addressing student fears about nurses and other healthcare providers in PPE (see figure 4). Mary Coldwell and Craig Matthes are encouraging their students through social media, letting them know they are missed and encouraging them to practice self-care (images on cover). State school nurse organizations are holding virtual town halls to connect with members, in some instances on a weekly basis. (image on cover)

### Called to Service in the Larger Community

**Figure 5.**
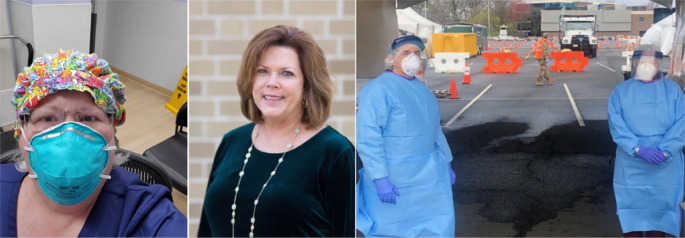
Andrea Ferguson; Eileen Gavin; Anthony Torres and Barbara Maher

Many of our colleagues have answered the call to serve the larger community, working on the front lines. Andrea Ferguson is working in the emergency room of her local hospital (Figure 5). Barbara Maher and Anthony Torres are staffing a drive through COVID-19 test site (Figure 5). Some school nurses, employed by hospital systems or health departments, are redeployed to other practice areas, necessitating self-evaluation of competence for the assignment and collaboration with employers to establish safe patient care environments.

Eileen Gavin, in collaboration with her health department, is training fellow school nurses to conduct contact tracing, a natural fit for school nurse public health practitioners (Figure 5). While working with one new mom, Eileen was able to arrange delivery of infant formula, saving the mother and baby a risky trip into the community.

### Physical Distancing and Stay at Home Orders

This new paradigm of working from home presents possibilities and challenges. There can be time to research unique student health conditions, collaborate with pediatricians and other healthcare providers, and update Individualized Healthcare Plans without the interruptions so common in a school health office. Perhaps you have found space in your day to carefully analyze your health office data, preparing a presentation for your administrator about your work both before and during school closing. Because many schools are relying heavily on school nurse expertise to help them navigate this crisis, more than a few school nurses administrators have expressed that they are working longer hours than ever to meet the needs of their school community.

We have learned that managing work and family responsibilities within the same space and time can be stressful. You may have children at home who are used to a schedule, while your need to balance work and childcare make a schedule difficult to achieve. Are you like me, juggling multiple curbside grocery pick-up services, trying to stock up on the essentials and favored items on a schedule that keeps your diet healthy and your family happy? You may be trying to safely care for older parents, or an immunocompromised family member and are fearful for their health when you must venture out into the community. Perhaps you have made the decision to brave the frontline of this pandemic and isolate yourself from family because you are uncertain of your COVID-19 status. Others of you may be experiencing job loss and uncertain futures.

### A Seat at the Table: Paving the Way

In speaking with Imo Jean Douglas (personal conversation, April 30, 2020) about these opportunities and challenges, she shares that this pandemic has garnered her a seat at the Executive Team table. Yet, Imo Jean is clear that it was the work she and her staff did prior to this crisis that paved the way. She looked to health data and the stories of students to demonstrate the necessity of school nurses to budget conscious administrators. She alerted her administrators early and often that COVID-19 was coming and that they must be prepared. She continues to compile data and stories from this season of pandemic to advocate that she retain her seat at the table.

NASN is paving the way for students access to school nurses. In late April, I sent a letter to President Trump (NASN 2020b), alerting him that it is imperative that students have access to a school nurse when schools reopen. This letter outlines for the President the essential role school nurses have in addressing the ongoing impact of the COVID-19 pandemic and informing plans to reopen the nation’s schools. We are asking that the White House fund 10,000 school nurses to conduct infectious disease surveillance, identification, and intervention for student physical and mental health concerns, health screenings, school-located vaccinations, and immunization compliance efforts. We know that keeping students in school will have tremendous implications for the economy, workforce, and families. Our call for stakeholders to sign on to a petition to the President has garnered almost 13,000 signatures in 9 days.

**Figure 6.**
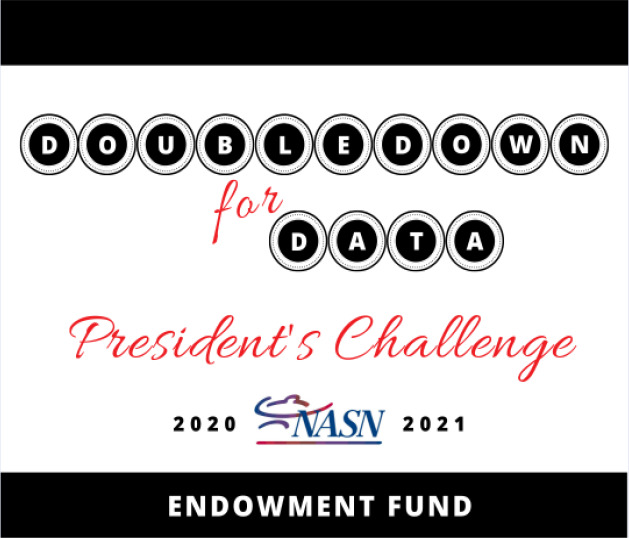
Doubledown for Data

You can help pave the way by contributing to NASN’s ability to fund school health and school nurse research, research that can better define the value we bring to student health and learning. Once again I want to ask that you support the Doubledown for Data President’s Endowment Fund challenge (Figure 6). No donation is too small—$2, $20, $200, or double last year’s donation. You can donate to the NASN Endowment Fund at https://my.nasn.org/donate-now\

## The Framework for 21st Century School Nursing Practice in Action

School nurses across the United States and overseas are bringing the Framework for 21st Century School Nursing Practice (NASN, 2016) to life. I hope that you recognize the Framework and its interrelated components of Standards of Practice, Leadership, Community/Public Health, Care Coordination, and Quality Improvement in the work that you do and in the stories of our school nurse colleagues. I hope that you are living into this model that guides school nursing practice (NASN, 2018) as we grow together in service to students, families, and communities. I urge you to use the Framework to articulate your school nurse practice to stakeholders (NASN, 2018), to provide a roadmap for continuous improvement, and a structure for meaningful school nurse self-reflection and performance appraisal (Allen-Johnson, 2017; Maughan et al., 2016; Wallin & Rothman, 2019). You will learn more about the Framework from two additional articles included in this issue of *NASN School Nurse*:

- Framework for 21st Century School Nursing Practice: Clarifications and Updated Definitions
- Care Coordination: A Principle of 21st Century School Nursing Practice with a Focus on Case Management

### Beyond a Pandemic: Recovery

I wonder what the course of COVID-19 will be by the time you read this message. I wonder how our lives and the lives of students and families will be changed. Will the expectation of increased mental health concerns in the school population be a reality? Are schools and the larger community equipped to meet these increased needs?

The promise of these events is that we are learning news ways to communicate with students and families—ways that I think will be good to continue beyond COVID-19. NASN and its Affiliate Organizations are certainly learning new ways to conference, communicate, advocate, and tap into the expertise of members.

I want to encourage you to focus on self-care, remembering that you must be well to bring others to wellness. You have made me proud to be a school nurse alongside you and I look forward to the remainder of our journey. Remember that we are #InItTogether.

With gratitude, 

*Laurie*